# Modern Pathology-Driven Strategies in Neoadjuvant Immunotherapy for Head and Neck Squamous Cell Carcinoma: From Residual Tumor Quantification to Spatial and AI-Based Biomarkers

**DOI:** 10.3390/cancers18061020

**Published:** 2026-03-21

**Authors:** Annabella Di Mauro, Rossella De Cecio, Saverio Simonelli, Margherita Cerrone, Rosalia Anna Rega, Maria Luisa Marciano, Monica Pontone, Imma D’arbitrio, Francesco Perri, Gerardo Ferrara

**Affiliations:** 1 Pathology Unit, INT-IRCCS—Fondazione G. Pascale, 80131 Naples, NA, Italy; r.dececio@istitutotumori.na.it (R.D.C.); saverio.simonelli@istitutotumori.na.it (S.S.); margherita.cerrone@istitutotumori.na.it (M.C.); rosalia.rega@istitutotumori.na.it (R.A.R.); imma.darbitrio@istitutotumori.na.it (I.D.); gerardo.ferrara@istitutotumori.na.it (G.F.); 2 Head and Neck Oncology Unit, INT-IRCCS-Fondazione G. Pascale, 80131 Naples, NA, Italy; ml.marciano@istitutotumori.na.it (M.L.M.); m.pontone@istitutotumori.na.it (M.P.); f.perri@istitutotumori.na.it (F.P.)

**Keywords:** head and neck squamous cell carcinoma, neoadjuvant therapy, immune checkpoint inhibitors, residual viable tumor, pathological response

## Abstract

Neoadjuvant strategies in head and neck squamous cell carcinoma (HNSCC) are reshaping therapeutic paradigms by shifting emphasis from anatomical staging toward biology-driven response stratification. The transition from induction chemotherapy to immune checkpoint–based and combination regimens has transformed the perioperative setting into a translational platform that enables interrogation of tumor–immune interactions and clonal selection under therapeutic pressure prior to surgery. In this context, pathological response assessment has emerged as a robust surrogate endpoint, overcoming the limitations of radiologic evaluation, which often fails to capture immune-mediated pseudoprogression and spatially heterogeneous regression. Quantification of residual viable tumor (RVT) provides a reproducible metric of therapeutic efficacy, while characterization of immune-related regression beds, tertiary lymphoid structures, macrophage polarization states, and compartment-specific nodal responses offers mechanistic insight into tumor clearance and resistance evolution. Evidence from phase II trials, single-cell sequencing, spatial transcriptomics, and multiplex immune profiling supports the prognostic relevance of pathology-driven endpoints. Integration of digital pathology and artificial intelligence–assisted image analysis further enhances reproducibility and enables high-resolution mapping of residual disease and immune architecture. Within this modern oncologic framework, the neoadjuvant-treated specimen functions as a dynamic biomarker platform guiding response-adapted surgical strategies and biomarker-driven clinical trial design. This study was designed as a narrative review. A structured literature search was performed using PubMed and major oncology journals to identify relevant studies on pathology-driven response assessment in neoadjuvant-treated head and neck squamous cell carcinoma. The review focused on publications addressing histopathological response criteria, immune microenvironment remodeling, spatial profiling technologies, and computational pathology approaches.

## Simple Summary

Oral cancer is often treated with surgery, but new strategies are exploring treatments given before surgery to shrink the tumor and stimulate the immune system. In this setting, traditional imaging does not always accurately show how much cancer remains. Careful examination of the removed tissue under the microscope provides more reliable information about treatment effectiveness. This review explains how pathologists can measure the amount of residual cancer cells and recognize immune-related changes within the tumor bed. We also discuss how modern technologies, such as digital pathology and molecular profiling, can improve response evaluation and help guide personalized treatment decisions. By standardizing how treatment response is assessed, clinicians may better identify patients who can safely receive less aggressive therapy and those who need additional treatment. This approach aims to improve survival while reducing unnecessary side effects in patients with oral cancer.

## 1. Introduction

Head and neck squamous cell carcinoma (HNSCC), including oral cavity squamous cell carcinoma (OSCC), remains a major burden in oncologic pathology and represents one of the most challenging malignancies in terms of locoregional control and treatment-related morbidity. In resectable locally advanced disease, prognosis is still largely dictated by adverse histopathological features and nodal risk factors that guide postoperative risk stratification and adjuvant treatment decisions [1,2]. Despite advances in surgical techniques and multimodal therapy, locoregional recurrence and treatment-related morbidity remain significant challenges in this patient population.

It is important to acknowledge that human papillomavirus (HPV)-positive oropharyngeal squamous cell carcinoma (OPC) represents a biologically distinct subset of HNSCC, characterized by a more favorable prognosis, a unique immune microenvironment, and increased sensitivity to systemic therapies, including immunotherapy. Although the present review primarily focuses on OSCC, where pathology-driven response assessment is particularly critical for surgical decision-making, emerging evidence suggests that HPV-positive OPC may exhibit distinct patterns of pathological response following neoadjuvant treatment [3,4].

Historically, neoadjuvant (induction) chemotherapy was explored with the aim of tumor downstaging, facilitating surgical resection, and eradicating occult systemic disease. However, randomized trials and subsequent meta-analyses failed to demonstrate a consistent overall survival advantage compared with upfront surgery, resulting in limited adoption of preoperative chemotherapy in routine clinical practice [2,5]. These early strategies were characterized by substantial heterogeneity in patient selection, treatment protocols, and response assessment methods, limiting the comparability and clinical applicability of results. From a pathology-driven perspective, a major limitation of early neoadjuvant programs was the lack of standardized tissue-based surrogate endpoints capable of accurately reflecting therapeutic efficacy. Radiological response criteria frequently fail to capture residual viable tumor and regression beds at both the primary site and cervical lymph nodes, particularly in the presence of therapy-induced inflammatory changes or stromal remodeling. In contrast, histopathological assessment provides direct insight into the biological effects of treatment and offers superior prognostic information. Indeed, tumor regression grade and the quantification of residual viable tumor have emerged as independent predictors of clinical outcome in neoadjuvant-treated oral cavity carcinoma cohorts [6,7,8].

The advent of immune checkpoint inhibitors has renewed interest in perioperative treatment strategies. Neoadjuvant immunotherapy can induce major pathological responses and reshape the tumor microenvironment even in the absence of marked radiological shrinkage, reflecting immune-mediated tumor clearance rather than simple tumor size reduction [9,10,11]. Recent phase II trials in resectable HNSCC and OSCC have confirmed the feasibility and safety of PD-1–based neoadjuvant approaches and have highlighted the central role of pathological assessment in response quantification and harmonized reporting [12,13,14]. These studies have further emphasized the importance of integrating morphological evaluation with immune and molecular biomarkers. This integrated approach enhances the understanding of treatment sensitivity and resistance mechanisms.

Within this evolving therapeutic landscape, the surgical specimen obtained after neoadjuvant therapy represents a unique biological archive that captures dynamic interactions between tumor cells, immune components, and stromal elements. Detailed histopathological evaluation of treated tumors therefore provides not only quantitative information on residual disease but also mechanistic insight into therapy-induced tumor regression and microenvironmental remodeling [15].

The aim of this review is to provide a comprehensive, pathology-centered synthesis of neoadjuvant treatment strategies in oral cavity and head and neck squamous cell carcinoma. Specifically, this review focuses on (i) standardized histopathological response assessment, with particular emphasis on residual viable tumor (RVT) as a clinically relevant surrogate endpoint; (ii) immune-related regression patterns and tumor microenvironment remodeling; and (iii) emerging spatial and computational biomarkers that may support response-adapted clinical decision-making [16]. This review does not aim to provide a systematic meta-analysis of therapeutic efficacy but rather to critically integrate current evidence from pathology, immuno-oncology, and computational approaches in order to define a biologically informed framework for treatment response evaluation in neoadjuvant settings. Importantly, this pathology-centered framework not only refines response assessment but also enables a paradigm shift toward biology-driven surgical decision-making, where treatment intensity may be adapted based on the quality and spatial distribution of residual disease rather than conventional staging alone. From a clinical and translational standpoint, this shift is particularly relevant because neoadjuvant therapy creates a time window in which treatment-induced biological changes can be directly interrogated in the resection specimen. Unlike the conventional postoperative setting, where pathology mainly serves prognostic stratification, the neoadjuvant context allows the pathologist to evaluate how a tumor has biologically responded under therapeutic pressure. This includes not only the extent of residual viable tumor, but also the quality of stromal remodeling, the distribution of immune infiltrates, and the persistence of resistant tumor niches. Accordingly, the surgical specimen becomes more than a static endpoint: it functions as an in vivo readout of treatment sensitivity and resistance, with immediate implications for biomarker development and response-adapted management.

### 1.1. Evolution of Neoadjuvant Strategies in Head and Neck Cancer

Early neoadjuvant approaches in head and neck squamous cell carcinoma (HNSCC) were primarily centered on induction chemotherapy, with the clinical goals of tumor downstaging, regional nodal control, and eradication of micrometastatic disease. However, randomized trials and subsequent meta-analyses failed to demonstrate a consistent overall survival benefit compared with upfront surgery, leading to a progressive decline in the use of neoadjuvant chemotherapy for resectable disease [2,5,17,18,19,20,21]. From a pathology-driven perspective, these early setbacks reflected not only limited therapeutic efficacy but also major methodological shortcomings, including heterogeneous patient selection and the absence of standardized tissue-based surrogate endpoints [5,6,7]. Subsequent pathology-focused studies demonstrated that histopathological parameters, tumor regression grade (TRG), residual viable tumor (RVT), nodal response, and margin status, carry significant prognostic value, establishing pathological response as a biologically meaningful endpoint [5,6,7,8]. These observations confirmed that radiologic response alone is an unreliable surrogate of true tumor clearance, as imaging frequently fails to capture regression beds and scattered residual microfoci, particularly within regional lymph nodes [5,6,17,18]. The clinical integration of immune checkpoint inhibitors (ICIs) has led to a renewed interest in neoadjuvant strategies. HNSCC is now recognized as an immunologically active malignancy characterized by a dynamic tumor microenvironment (TME) that modulates therapeutic response [19,20]. Translational evidence suggests that neoadjuvant immunotherapy preserves in situ tumor–immune interactions, facilitating antigen presentation and systemic immune priming—biological processes that are attenuated following upfront surgical resection [21,22,23].

Recent single-cell and spatial analyses have further clarified that immune-featured stromal niches, macrophage polarization programs, and spatial immune organization are strongly associated with treatment sensitivity or resistance [8,9]. Collectively, these findings reinforce the concept that the resection specimen provides unique mechanistic insight into treatment effects. Beyond RECIST-based shrinkage, pathological examination captures immune-mediated regression beds, fibro-inflammatory remodeling, and compartment-specific RVT, offering a biologically refined assessment of therapeutic impact. Beyond these morphological features, increasing attention has been directed toward the characterization of the tumor immune microenvironment, which provides additional functional insight into treatment response.

### 1.2. Pathological Response Assessment After Neoadjuvant Therapy

The evaluation of therapeutic efficacy in HNSCC has progressively shifted from a radiology-centered model to a pathology-driven framework. In the neoadjuvant setting, the surgical specimen represents the definitive platform for integrating quantitative and qualitative measures of response, enabling direct assessment of treatment-induced tumor and stromal changes. Conventional radiological criteria, although essential for staging, frequently fail to discriminate residual viable tumor (RVT) from therapy-induced fibro-inflammatory regression beds. This limitation is particularly evident following immune-based regimens, where imaging may demonstrate apparent tumor persistence or enlargement (“pseudoprogression”) despite substantial pathological clearance. In contrast, histopathological evaluation provides superior prognostic stratification and mechanistic insight into treatment response [24,25,26]. From a clinical standpoint, RVT-based pathological response categories are increasingly being evaluated as operational tools for postoperative stratification in OSCC. Patients achieving major pathological response or minimal residual viable tumor may represent potential candidates for treatment de-escalation strategies within clinical trials, including reduction in adjuvant therapy intensity or modification of surgical approaches. Conversely, the persistence of substantial RVT may identify biologically resistant disease and support the use of intensified adjuvant therapy or enrollment in escalation trials. In this context, pathology-driven response assessment provides a practical bridge between tissue-level biological information and multidisciplinary therapeutic decision-making.

Within contemporary multidisciplinary management of oral cavity carcinoma, the role of the pathologist has consequently expanded beyond conventional staging. Neoadjuvant-treated specimens allow assessment of residual tumor burden, characterization of immune-related regression patterns, and identification of resistance-associated stromal niches. These tissue-based parameters increasingly inform risk stratification and may influence decisions regarding surgical modulation and adjuvant intensification strategies. Beyond quantitative assessment of residual viable tumor, increasing attention has been directed toward the biological characterization of the tumor microenvironment, which provides additional insights into treatment response. In this context, RVT assessment may serve as a critical decision-making node within multidisciplinary tumor boards, enabling real-time integration of pathological response with surgical planning and adjuvant treatment selection. These observations further support the incorporation of RVT as a standardized surrogate endpoint in future neoadjuvant clinical trials.

### 1.3. Residual Viable Tumor (RVT) as the Core Quantitative Endpoint

Across chemotherapy-based series and contemporary immunotherapy trials, quantification of residual viable tumor (RVT) within the treated tumor bed has emerged as the most reproducible and clinically meaningful pathological endpoint. Early OSCC cohorts demonstrated that regression grade and RVT, rather than radiological response, independently predict survival and locoregional control [5,6,17].

In the era of neoadjuvant immunotherapy and chemo-immunotherapy, treatment-induced inflammatory expansion, stromal edema, and architectural distortion frequently confound imaging-based evaluation. RVT-centered pathological assessment, by contrast, provides a biologically grounded measure of true tumor clearance and correlates more consistently with clinical outcomes [27,28,29]. Accordingly, RVT-based response categories, including pathological complete response (pCR), major pathological response (mPR, ≤10% RVT), and partial or non-response, are increasingly incorporated into OSCC-specific neoadjuvant protocols. These classifications promote harmonized reporting, improve inter-trial comparability, and facilitate the use of pathological response as a surrogate endpoint in translational studies. Beyond its quantitative value, RVT also offers practical advantages for routine pathology reporting. It can be applied across different therapeutic backbones, facilitates communication within multidisciplinary teams, and provides a common language for comparing chemotherapy-based, immunotherapy-based, and combined neoadjuvant regimens. Importantly, RVT assessment is not limited to identifying complete responders; it also captures intermediate biological states that may be clinically meaningful, such as major pathological response with focal resistant residual nests or partial response with extensive stromal regression. This gradation is particularly relevant in OSCC, where anatomical complexity, nodal burden, and the morbidity of adjuvant escalation require more nuanced postoperative decision-making than a simple binary responder/non-responder classification [25,28,30,31].

### 1.4. Immune-Related Regression Beds and Therapy-Induced Stromal Remodeling

Neoadjuvant immunotherapy induces distinctive immune-related regression beds (IRRBs), defined as areas of therapy-induced tumor clearance characterized by fibrosis, inflammatory infiltrates, neovascularization, and immune activation reflecting prior tumor involvement. These immune-mediated architectural patterns differ substantially from the confluent infarct-type necrosis typically observed after cytotoxic chemotherapy. Accurate recognition of IRRBs is critical to avoid misclassification of treatment response and underestimation of tumor regression in resected HNSCC specimens [25,28,30,31].

Emerging translational and spatial profiling studies demonstrate that pathological responders harbor organized immune-featured stromal niches composed of spatially structured CD8^+^ T-cell infiltrates, functionally polarized macrophage subsets, and activated cancer-associated fibroblasts. These microenvironmental configurations correspond morphologically to immune-rich regression beds and are consistently associated with favorable therapeutic outcomes [8,9,32,33].

Collectively, these findings support the concept that the treated surgical specimen provides not only quantitative information on residual viable tumor, but also qualitative insight into immune-mediated tumor eradication and stromal reprogramming. Systematic histopathological characterization of these patterns is therefore essential for standardized response assessment in oral cavity and head and neck squamous cell carcinoma.

#### 1.4.1. Spatial Heterogeneity and Mapping of Regression Patterns

Whole-specimen evaluation and large-format tumor bed mapping demonstrate that pathological regression in HNSCC is frequently spatially heterogeneous. Rather than exhibiting uniform centripetal contraction, treated tumors often display a discontinuous “starry-sky” pattern, characterized by scattered microfoci of residual viable carcinoma (RVC) embedded within therapy-altered stroma. These residual nests are frequently radiologically occult and may be missed without systematic, extensive sampling of the entire tumor bed and resection margins [27,29,34].

**Figure 1:**
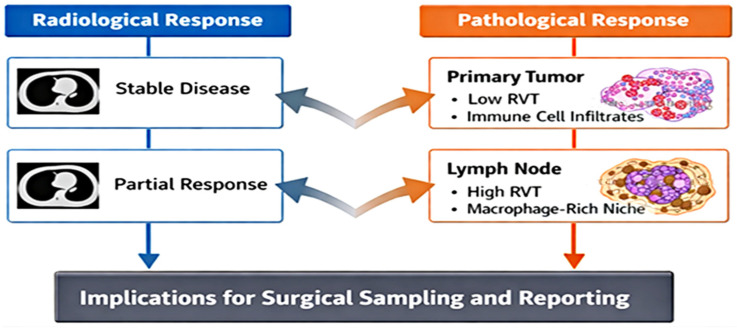
Neoadjuvant Immunotherapy Uncouples Imaging from True Tumor Clearance. Neoadjuvant immunotherapy decouples radiologic tumor size from biological response. Imaging may suggest persistent disease due to immune-cell infiltration and stromal remodeling, while histopathology demonstrates minimal residual viable tumor within immune-rich regression beds. Accurate response classification therefore depends on standardized pathology-based assessment rather than radiologic shrinkage alone.

Radiological–pathological discordance is particularly evident following neoadjuvant immunotherapy. Treatment-induced inflammatory expansion, stromal edema, and dense immune infiltrates may simulate persistent disease on imaging (“pseudoprogression”), whereas histopathological examination reveals substantial tumor regression. This discordance underscores the necessity for standardized grossing protocols, meticulous tumor bed mapping, and strict radiologic–pathologic correlation to ensure accurate response classification [25,27,28,34]. A schematic representation of radiologic–pathologic discordance after neoadjuvant therapy is shown in Figure 1.

Building upon these morphological and spatial features, increasing attention has been directed toward dynamic immune and stromal biomarkers that further refine response characterization.

#### 1.4.2. Primary Tumor Versus Nodal Disease: Compartmental Heterogeneity

Pathological response assessment following neoadjuvant therapy should be performed independently for the primary tumor and cervical lymph node metastases, as discordant compartment-specific responses are frequently observed. Lymph node metastases may retain substantial residual viable tumor (RVT) despite marked regression at the primary site, whereas in other cases nodal deposits may demonstrate deeper immune-mediated clearance. This compartmental dissociation supports the concept that nodal disease exhibits partial biological and microenvironmental independence from the primary tumor [34,35,36].

Beyond quantitative RVT differences, qualitative microenvironmental disparities are increasingly recognized. Translational studies indicate that metastatic lymph nodes may harbor macrophage-enriched, immunosuppressive stromal niches capable of sustaining localized resistance programs. Such niches are characterized by distinct macrophage polarization patterns and altered immune–stromal crosstalk, which may explain selective nodal persistence despite apparent primary tumor response [19,32,37,38].

From a surgical pathology standpoint, these observations mandate separate and explicit reporting of primary and nodal response. Nodal regression should be documented as an independent endpoint, incorporating compartment-specific RVT quantification, characterization of immune-related regression beds, and identification of resistance-associated stromal patterns. This compartmentalized approach strengthens risk stratification and improves the biological interpretation of neoadjuvant treatment effects in oral cavity and head and neck squamous cell carcinoma. This distinction is clinically relevant because discordant primary-versus-nodal responses may otherwise be obscured by global response summaries. A patient with marked regression at the primary site but persistent viable nodal disease may still harbor a biologically aggressive compartment that justifies intensified postoperative surveillance or adjuvant treatment. Conversely, substantial nodal clearance in the setting of limited primary-site regression may suggest differential compartmental sensitivity rather than generalized resistance. These scenarios illustrate why compartment-specific pathology reporting should be considered an integral part of modern neoadjuvant assessment rather than an optional descriptive addendum.

## 2. Dynamic Immune and Stromal Biomarkers in Treated Specimens

Comparative analysis of matched pre-treatment biopsies and post-neoadjuvant resection specimens demonstrates that therapy dynamically remodels the tumor microenvironment. Shifts in PD-L1 expression (Combined Positive Score, CPS) and variations in tumor-infiltrating lymphocyte (TIL) density are consistently observed following immune-based regimens. The surgical specimen therefore represents a critical diagnostic window for reassessing these biomarkers and documenting treatment-induced immune plasticity not captured by baseline evaluation [22,39,40,41,42].

**Figure 2:**
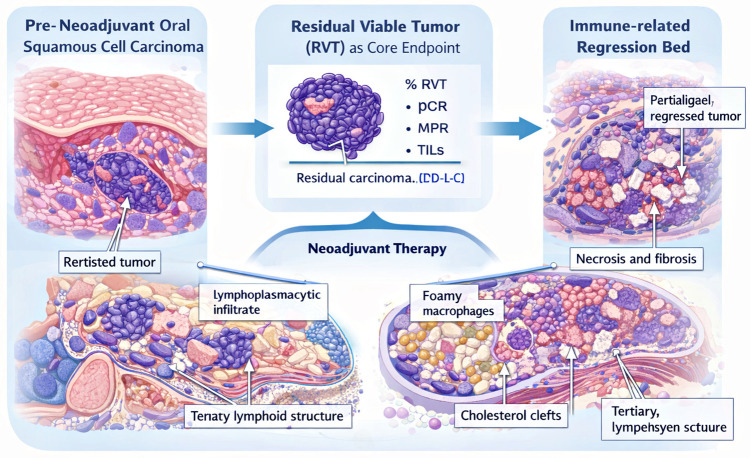
Standardized Pathology-Driven Framework for Response Assessment after Neoadjuvant Therapy. Comprehensive evaluation of neoadjuvant-treated specimens requires quantitative assessment of residual viable tumor (RVT), recognition of immune-related regression beds, reassessment of dynamic biomarkers (PD-L1, TIL density), and independent analysis of primary tumor and nodal compartments. Integration of morphologic, immune, and spatial parameters supports response-adapted multidisciplinary management and improves inter-trial comparability.

Beyond immune checkpoint modulation, neoadjuvant therapy alters additional stromal and metabolic components, including macrophage composition, fibroblastic activation, and vascular remodeling. These changes highlight the treated resection specimen as an essential platform for integrated biomarker assessment and identification of resistance-associated niches [43,44,45,46,47,48]. The key histopathological parameters required for standardized response assessment in oral cavity squamous cell carcinoma are summarized in Figure 2.

### 2.1. Pathology-Driven Response-Adapted Management

Systematic quantification of residual viable tumor (RVT), combined with recognition of immune-related regression beds and compartment-specific nodal responses, provides a reproducible tissue-based framework for postoperative risk stratification and individualized surgical modulation [20,21,35,46,47,48,49,50]. In selected cases demonstrating deep pathological response, reduction in surgical morbidity or avoidance of unnecessary adjuvant intensification may be considered within structured clinical protocols.

Conversely, persistence of substantial RVT or identification of unfavorable stromal and immune regression patterns defines biologically resistant disease. These features frequently correlate with incomplete immune activation, macrophage-dominant suppressive niches [37,38], or spatially heterogeneous residual carcinoma, suggesting an increased risk of locoregional relapse. Such findings may justify adjuvant treatment intensification, integration of systemic immunotherapy, or enrollment in escalation clinical trials designed for high-risk molecular or pathological subsets [30,31,36,48,51]. Collectively, pathology-defined response assessment is emerging as a central surrogate endpoint in contemporary neoadjuvant protocols, directly informing multidisciplinary decision-making, surgical planning, and biomarker-driven trial design (Table 1).

From a clinical perspective, the quantification of residual viable tumor (RVT) has direct implications for postoperative management. Patients achieving major pathological response or near-complete tumor regression may be candidates for treatment de-escalation strategies, potentially avoiding unnecessary adjuvant therapies and associated toxicity. Conversely, the presence of substantial RVT or discordant nodal response may identify high-risk patients who could benefit from treatment intensification or enrollment in clinical trials. In this context, pathology-driven response assessment is emerging not only as a prognostic tool but also as a clinically actionable biomarker guiding personalized therapeutic decision-making.

### 2.2. Future Perspectives: Spatial Biology, AI and Integrated Biomarkers

Neoadjuvant-treated surgical specimens represent dynamic biological archives that extend far beyond conventional residual viable tumor (RVT) quantification. The integration of morphology with immune architecture (i.e., the spatial organization and distribution of immune cells within tumor and stromal compartments), stromal remodeling, and spatially resolved molecular profiling is progressively redefining pathological response assessment in oral cavity squamous cell carcinoma (OSCC) [39,40,44].

Recent advances in spatial transcriptomics and multiplex immune profiling enable high-resolution mapping of immune-activated versus immune-excluded tumor ecosystems within regression beds. Spatially resolved platforms allow identification of localized resistance programs within residual viable tumor foci, including interferon-signaling gradients, checkpoint ligand expression, cytokine networks, and stromal–immune cross-talk [22,39,44,45]. These approaches transform the regression bed from a static fibrotic scar into a biologically informative biomarker landscape.

Composite immune signatures integrating PD-L1 expression dynamics, tumor-infiltrating lymphocyte (TIL) density, interferon-γ–related gene programs, macrophage polarization markers, and tertiary lymphoid structure organization may refine response categorization beyond simple RVT thresholds [33,34,44,45]. Immune-featured regression beds characterized by organized CD8^+^ T-cell infiltrates and activated fibroblastic niches consistently correlate with major pathological response, whereas macrophage-enriched immunosuppressive microenvironments are frequently associated with persistent RVT and nodal discordance [37,40].

While immune-related biomarkers provide functional insight into tumor response, their integration with computational pathology approaches enables a more comprehensive and quantitative evaluation of spatial tumor–immune interactions. Recent efforts toward multimodal data integration and machine learning–driven precision oncology in head and neck cancer further underscore the potential of combining histopathology, clinical variables, and immune profiling into predictive frameworks [52].

Artificial intelligence–assisted digital pathology further strengthens this framework by enabling objective quantification of RVT, spatial immune gradients, and stromal remodeling patterns. Integration of AI-derived morphologic features with transcriptomic and immune biomarkers may enable clinically actionable models for preoperative identification of resistant phenotypes and for response-adapted therapeutic strategies [28,52,53,54]. Advances in targeted drug-delivery systems, including tumor-targeting liposomal platforms, may further enhance therapeutic precision and complement biomarker-guided treatment strategies in precision oncology [55]. A further priority for future studies will be the prospective integration of pathology-derived metrics into adaptive trial designs. In this setting, RVT, regression-bed architecture, and compartment-specific nodal response could be incorporated as intermediate decision points for treatment escalation, de-escalation, or biomarker-enriched patient selection. The real value of artificial intelligence and spatial profiling will likely emerge when these tools are not used in isolation, but embedded within clinically annotated, prospectively collected datasets that link morphology, immune states, and outcome. Such an approach may help move the field beyond descriptive associations toward operational models capable of supporting real-world therapeutic decisions. Future research priorities include:

- Prospective validation of composite morphology–immune–molecular response scores as survival surrogates;
- Standardization of spatial and digital quantification workflows in neoadjuvant OSCC protocols;
- Integration of AI-derived morphometric metrics with transcriptomic signatures into predictive nomograms;
- Functional targeting of resistance-associated microenvironmental niches identified within residual viable tumor areas.

Standardization of pathology-driven response assessment may represent a paradigm shift comparable to the introduction of AJCC staging in surgical oncology [56]. Ultimately, the convergence of spatial biology, artificial intelligence, and pathology-driven metrics is expected to redefine response assessment as a dynamic, multi-layered biomarker system capable of capturing both tumor-intrinsic and microenvironmental determinants of therapeutic outcome.

## 3. Conclusions

Neoadjuvant therapy is progressively reshaping the management of resectable oral cavity squamous cell carcinoma (OSCC) by shifting emphasis from purely anatomical staging toward biologically informed response stratification. Within this evolving paradigm, pathology-based assessment centered on residual viable tumor (RVT) has emerged as the most robust and reproducible surrogate endpoint, consistently demonstrating superior prognostic relevance compared with radiologic evaluation and enabling inter-trial harmonization of response criteria [25,26,27,28,29,48,49,50].

Systematic quantification of RVT, integrated with recognition of immune-related regression beds and compartment-specific assessment of primary tumors and cervical lymph nodes, provides a standardized framework for response classification and postoperative risk stratification [20,21,25,28]. Increasing evidence indicates that the treated surgical specimen captures therapy-induced immune remodeling, macrophage polarization states, tertiary lymphoid organization, and resistance-associated stromal niches that are not discernible through imaging alone [37,40,44,45]. These tissue-based features refine biological interpretation of treatment efficacy and directly inform multidisciplinary decision-making in the perioperative setting [30,31,36].

As spatially resolved immune profiling and digital pathology platforms continue to mature, pathological response assessment is evolving from a static percentage-based metric into a multidimensional biomarker system. Integration of morphologic scoring with immune architecture, transcriptomic gradients, and computational image analysis enables more precise identification of responders and resistant phenotypes [28,39,40,53]. In this context, the resection specimen becomes a central integrative platform capable of guiding response-adapted de-escalation in patients achieving major pathological response, while supporting rational escalation strategies in biologically resistant disease.

Collectively, the integration of standardized pathological scoring, spatial biology, and computational analytics establishes a unified framework for biology-driven patient stratification in neoadjuvant OSCC. In this framework, pathology transcends its traditional diagnostic role, emerging as a central, integrative driver of precision oncology, where the surgical specimen functions as a dynamic decision-support platform linking tumor biology, therapeutic response, and clinical outcome.

## Figures and Tables

**Table 1:** Pathology-driven framework for response assessment after neoadjuvant therapy in oral cavity squamous cell carcinoma.

Domain	Pathological Parameters to Assess	Reporting Standard	Key Diagnostic Pitfalls
Residual viable tumor (RVT)	Percentage of viable carcinoma within treated tumor bed	Exact percentage + response category (pCR, mPR ≤ 10%, PR, NR)	Keratin granulomas; therapy-related atypia mimicking carcinoma
Regression bed	Immune-rich vs. necrotic vs. fibrotic patterns; stromal remodeling	Qualitative description of dominant regression phenotype	Misinterpretation of inflammation or fibrosis as residual tumor
Spatial pattern	Centripetal vs. scattered (“starry-sky”) residual foci	Tumor bed mapping with macro–micro correlation	Under-sampling of treated tumor bed
Nodal compartment	RVT and regression patterns in metastatic lymph nodes	Separate assessment for each nodal basin	Discordant primary vs. nodal response
Immune biomarkers	PD-L1 (CPS); intratumoral and stromal TIL density	Pre- and post-treatment comparison when available	Dynamic modulation and CPS shifts
Resistance niches	Macrophage-rich or immunosuppressive stromal areas	Qualitative description ± targeted IHC	Under-recognition of resistant subclones

CPS, Combined Positive Score; IHC, Immunohistochemistry; mPR, major Pathological Response; NR, No Response; pCR, pathological Complete Response; PD-L1, Programmed Death-Ligand 1; PR, Partial Response; RVT, Residual Viable Tumor; TILs, Tumor-Infiltrating Lymphocytes.

## Data Availability

No new data were created or analyzed in this study.
